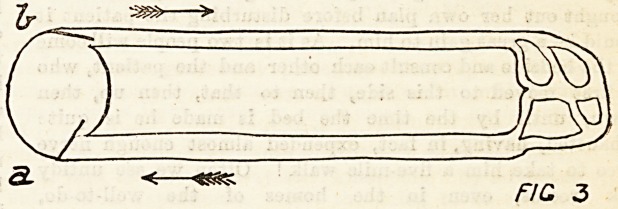# The Hospital Nursing Supplement

**Published:** 1895-08-24

**Authors:** 


### "The Hospital" Nursing Mirror.

Being the Extra Nursing Supplement of "The Hospital" Newspaper.

[Contributions for this Supplement should be addressed to the Editor, The Hospital, 428, Strand, London, W.C., and should have the word "Nursing" plainly written in left-hand top corner of the envelope.]

### News from the Nursing World.

### EMINENTLY PRACTICAL.

The preliminary training of pupil probationers for the London Hospital has made a most successful commencement, the first set of pupils being transferred last week from Tredegar House to the training school of the great East-end hospital. The practical aspect of the new scheme is peculiarly commendable, and deeply impresses itself on those inspecting the admirably-planned establishment in Bow Road. The second set of pupils were installed last Saturday, and there seems every prospect of these carefully selected candidates doing credit to the advantageous introduction to hospital work which Miss Lückes' scheme secures to them. This preliminary initiation into the uses, appearance, and technical names of things peculiar to hospitals removes from the path of the probationer many difficulties that have hitherto beset first days "on duty." Miss Lückes and the committee, by whom her administration in the nursing department is so consistently supported, may be congratulated on the new era in nurse training inaugurated at Tredegar House.

### ADDITIONS AND IMPROVEMENTS.

The new wards at the Western Fever Hospital are approaching completion, one being already in process of furnishing. They are beautiful rooms as regards light, air, and floor space, and the bay placed half way down each is an addition which answers the purpose of a day room for convalescents. This bay has an open fireplace with windows on either side, and easy chairs to give a home-like appearance. There is a table for adults to have their meals at, and a lower one to suit the smaller stature of the children. The ward kitchens are large and light, and each has a pantry on the coolest side of the new blocks of buildings. A recent addition to the comfort of the nursing staff has been made by a sitting-room set apart for the charge nurses, the staff and assistant nurses having already one of their own.

### "LEFT TILL CALLED FOR."

The conversion of a railway waiting-room into a temporary nursery for children suffering from eye affections was apparently looked upon with disfavour the other day at Liverpool Street, for an official is reported to have made it his business to return the young travellers to the Brentwood Schools. These poor little Hackney paupers had been sent in charge of a nurse to Moorfields Hospital, and it is stated that she left them in the waiting-room and went off to spend the day with her friends! The desertion of these afflicted children seems, according to the press report, to admit of neither excuse nor pardon. The Hackney Guardians, however, appear to hold unique opinions as to their own obligations, for they have merely temporarily suspended the so-called nurse. Presumably the children will therefore again be left to the tender mercies of a woman who has already shown herself unworthy of the Guardians' confidence.

### A SUCCESSFUL BAZAAR.

The brilliant bazaar in the grounds of Duff House, Banff, in aid of the Chalmers Hospital resulted in the total receipts reaching the handsome amount of over £1,230 by the close of the sale. The hospital stall was an exceptionally attractive one, presided over by the matron, Mrs. Gray, and a party of ladies. Members of the present nursing staff and several former workers also gave their services on the occasion. Many beautiful articles were conspicuous on this and the other stalls, and called forth much admiration from the Duke and Duchess of Fife, by whom, as recorded last week, the bazaar was formally opened. Mrs. Gray accepted the post of matron at Chalmers Hospital in 1880, and she has now a staff of six nurses, who are trained by her, theoretically and practically, for a period of three years, lectures being also given by the assistant surgeon.

### GIRL NURSEMAIDS.

"She took it into bed with her to pacify it," was the explanation reported as given by a young girl who recently awoke to find her mistress's baby dead by her side. "Accidental suffocation," was the verdict of the coroner's jury, although suggestions of cruelty had been made by the girl's fellow-servants and the baby's grandmother. However, the young servant was exonerated from all blame by the jury. It appeared from the press reports that this sixteen-year-old nursemaid had the baby by night as well as by day. It is hardly surprising that the poor infant was given to fretting; whilst the girl "was tired of nursing," having probably no special fitness for the work. Whilst most mistresses would hesitate to trust the cooking of food or the washing of silver and glass to so young a servant, many appear to consider that "she will do to look after the baby."

### DISTRICT NURSES FOR SOLIHULL.

The discussion as to whether a subscription should be made towards the provision of nurses for the various parishes of the Union by the Solihull Guardians, gave rise to various suggestions. A member of the Board considered it "morally impossible" to have trained nurses, but his reasons for this belief do not appear in the press reports of the recent meeting. He is said to have proposed that a list of two or three suitable women in each parish might be kept, and these "might acquire the requisite knowledge." It did not transpire whether district nurses were to be engaged whilst these "suitable" women underwent the course of proper training without which the Local Government Board will not be likely to sanction their employment under the Poor Law. The attendance of a fully-trained and competent woman, such as the Guardians have it in their power to secure, would prevent many persons from becoming burdens on the ratepayers. If the Solihull Guardians provided competent district nurses to work under the medical officer, they would find many lesser ailments of the poor could be effectually and economically treated in their own homes.

### THE ROYAL BRITISH NURSES' ASSOCIATION.

Special attention is called in the *Nurses' Journal* for August to the library now in process of formation at the Royal British Nurses' Association. Publishers seem to have responded liberally to Princess Christian's request for contributions; and a set of rules has been framed, to take effect at the opening of the library on October 1st. In regard to the educational lectures the treasurer reported that the association had lost about £20 on each course, and it was agreed at the general council meeting in July that these lectures should not be continued in this coming winter. The removal of 156 names was a step necessitated by the subscriptions of these members being four or five years in arrear.

### A TRAINED NURSE FOR DUNMOW.

At a meeting recently held at Dunmow Town Hall it was decided to form a Ladies' Committee and to engage a district nurse for the poor. An admirable speech was made by the Countess of Warwick, who presided at the meeting, and eloquently pleaded for the foundation of a Nursing Association for the district, and the engagement of a properly qualified nurse, who besides being a skilful and sympathising woman would be competent to teach the poor the best means of putting their houses into a sanitary condition. The position already taken by the Countess of Warwick as a Poor Law guardian and her advocacy of trained nursing alike afford good promise that a high standard of efficiency will be maintained in movements countenanced by her ladyship.

### THE NEW NURSE.

In the *Realm* for August 9th there appears a short article on "The New Nurse," which is only worth mentioning because it illustrates the ignorance and ingratitude with which some people judge those who have helped them in sickness. The author of this precious article says that he was "delivered over, at a private hospital, for the purposes of a small surgical operation, into a regiment of these sexless creatures." He is especially indignant because, when his hand accidentally touched that of the nurse while she was dressing his wound, she "in a pet" (!) made for the nearest basin that she might "wash off the pollution." So the nurse's reward for careful work is that this incarnation of ignorance writes that she, "being a perfect lady, shrinks, of course, from the sufferer's touch," and oddly enough, an editor is found to accept such stuff. Did neither of them ever hear of antiseptic surgery? What this gentleman expected he does not say; but his disappointment in his surroundings is thus expressed: "As was the furniture, warranted not to answer the purpose for which it was made, so were the mansion's proprietress and the attendant nymphs. No matronly caps with white ribbons; no semi-maternal attentions; but much of the New Woman bodily transported from the stage—hard as nails, about as gentle-eyed as a Gorgon. . . . Imagine a wing of Broadmoor peopled by giggling, unattractive grisettes arrayed in washed-out blue-print frocks, presided over by a grenadier in petticoats whom the doctor called 'Sister,' and platonically seemed to admire. You will thus have a fair idea of the New Nurse as I saw her, and of the new nursing system as I was its victim." The allusion to Broadmoor, where the patients are all insane, is happy, although hardly in the sense intended. In charity we must believe that the "small surgical operation" was a bigger thing than he thought, and that the writer has but penned the recollections of his delirium.

### PRECAUTIONS RESCINDED.

The readmission of patients' visitors to the sick wards of the Union was discussed at a recent meeting of the North Dublin Board of Guardians, and was approved by a majority. A proposal to take medical opinions regarding the health of the city, before letting people come in, three days every week met with but few supporters, although the advisability of consulting the dispensary doctors in such a matter seems obvious.

### ON THE LABRADOR.

An excellent supply of drugs has been provided for the two little hospitals on the Labrador by a party of Montreal ladies, headed by Miss Roddick. The steam yacht *Sir Donald* has also been stocked with drugs by their energy, and is cruising along the coast during the summer. The arrival of the doctors and nurses was most eagerly anticipated by the fishers and their families, who show each season how much they value their presence. The extreme destitution of the dwellers on the bleak Labrador coast greatly increases their sufferings. Supplies of warm clothing and nourishment form an essential part of the cargo of this ship, which has been sent out under the auspices of the Mission to Deep Sea Fishers.

### LEPERS IN THE EAST.

Seventeen asylums have been opened by the Mission to Lepers in India and the East, and there are many stations connected with this society in India, Burmah, and Ceylon. Missionaries associated with fifteen different societies co-operate in the work, which now includes the management of eight homes for the reception of the children of lepers. The aim of the Mission seems excellent, and is zealously carried out, but the missionaries report that many of the sufferers who ought to be in these asylums, for their own comfort and for the benefit of the community, object to enter because of the "Christian influence" which prevails there. Probably these prejudices only need time and experience to remove them.

### SHORT ITEMS.

It is announced in the local press that Mrs. Daly's resignation of her post of matron of the Plymouth Borough Hospital is consequent on her appointment as Chief Nurse in the Palace of the Ameer of Afghanistan.—A gift of £11 has been handed over to the Clitheroe District Nursing Association by the committee of the Cycle Costume Parade.—The District Nurse at Addlestone has paid 3,000 visits in eleven months.—A second trained nurse has been secured for the Hexham Workhouse Hospital. — The Portsea Guardians have appointed as nurse in the female imbecile wards Miss F. Laird, of Caine Hill Asylum.

### Elementary Physiology for Nurses.

By C. F. Marshall, M.D., B.Sc., F.R.C.S.

### III.—THE CIRCULATORY SYSTEM.

### The Blood Vessels.

As we said in the last lecture, the main purposes of the circulatory system are as follows: (1) To carry food from the digestive organs to all parts of the body; (2) to carry oxygen from the lungs to all parts of the body; (3) to carry carbonic

FIG I

H, heart; A, arteries; V, veins; C, capillaries.

acid from all parts of the body to the lungs, and other excretory matters to the organs which get rid of them; (4) to maintain the temperature of the body. The circulatory system consists chiefly of a set of tubes, distributed to all parts of the body and filled with blood. The arteries carry the blood to all parts of the body; the veins return the blood from the body; the heart is the pump or engine which receives blood from the veins and drives it into the arteries; the capillaries are very fine tubes, only visible under the microscope, connecting the terminal branches of the arteries with the commencements of the veins.

The course of the circulation in any part will be represented by the following diagram, in which the arrows show the direction of the flow of blood. Almost every part of the body is vascular, *i.e.*, contains blood vessels, and the closeness of the capillary network is difficult to realize. If we prick almost any part of our body it will bleed, showing that the vessels are so close together that there is not even room for the point of a needle between them. The non-vascular parts of the body, or those which have no blood-vessels, are the epidermis of the skin, the nails, hair, teeth, and cartilage.

The blood-vessels form a system of closed tubes, having no openings in their walls, so that everything that passes either into or out of the blood must pass through the walls of the

FIC 2

Diagram of Circulation.

LA, left auricle; RA, right auricle; LV, left ventricle; RV, right ventricle; L, capillaries of lungs; S, systemic capillaries.

vessels. The walls of the capillaries are very thin, and the passage of substances through them is easy. The walls of the veins and arteries are thick and tough, those of the arteries being highly elastic, like indiarubber, and stretching readily. But the capillaries are the most important part of the circulatory system, for in them the real work is done.

### The Heart.

This is the most important organ in the body. In shape it is conical, and about the size of one's fist. It lies in the chest with the large blood-vessels attached to its base. Its apex points forward and to the left, between the fifth and sixth ribs. The heart is really a dilated blood-vessel, as is seen by comparison with the hearts of the lower animals, in some of which it is simply a dilated muscular blood-vessel. The heart is divided into right and left halves by a median partition, each completely separate from the other, and each again divided transversely into two other cavities, an auricle and ventricle. We have therefore a right auricle and ventricle and a left auricle and ventricle.

### Course of the Circulation Through the Heart.

The right auricle receives blood returned by the veins from the body and sends it into the right ventricle. From this it is driven to the lungs. The left auricle receives blood returned from the lungs and sends it to the left ventricle, which sends it all over the body by the arteries.

The auricles have but little work to do, hence their walls are thin, and equal on both sides. The ventricles, on the other hand, have more work to do; hence their walls are

FIG 3

thick and muscular. As there is greater difficulty in moving the blood through the general system than through the lungs only, the left ventricle is much thicker and more muscular than the right.

### The Valves of the Heart.

We have said that the blood circulates, *i.e.*, flows round and round through the blood vessels, and always in the same direction, but we have not explained why this should be, or how it is effected.

Supposing we have a system of indiarubber pipes connected with an indiarubber ball and full of fluid. If we squeeze the ball it is as easy for the fluid to flow out one way as the other, but in order to ensure that the flow shall be in one direction only valves are necessary. These valves are flaps opening in one direction only, like the door of a room. Suppose we have one valve only, at *a*: On squeezing the ball we should empty it along the arteries only, but on letting go the ball a great part of the fluid would flow back again into the ball. If we have a second valve at *b*, on squeezing the ball, we equally send the fluid into the arteries; but when we relax the ball, and owing to its elasticity it dilates, it sucks the fluid from the veins only, and it is no longer possible for fluid to get back from the arteries to the heart, for the valves close instantly owing to the pressure of fluid on them.

### Dundee Royal Infirmary.

The annual distribution of prizes to successful nurses held in the board-room of the Royal Infirmary was attended amongst others by the chairman of the Board of Directors, Mr. William Kidd (who distributed the prizes) Mr. Halley, Mr. Lennie, and Mr. Anderson (directors), Dr. Nathan Raw, medical superintendent, and the Matron, Miss Strong. A very handsome volume bearing a suitable inscription was presented to each of the successful nurses whose names have already been inserted in the columns of the *Nursing Mirror*.

### Hints for Home Nursing.

### WANT OF THOUGHT.

Probably there is no profession that has been alternately praised and abused as much as that of sick nursing. On the one hand we hear nurses spoken of as self-denying angels, a band of noble women doing glorious work, unrecognised heroines, &c. On the other hand, and the cry gets louder as time goes on, we are met with, "I would not have a trained nurse in the house, not if I were dying! A set of self-opinionated women, who upset the whole establishment, and, except perhaps in the matter of surgical dressings, do little that we cannot do ourselves."

Probably there is something to be said on both sides, but does love take the place of knowledge, and do good intentions answer as well as training? "Evil is wrought for want of thought as well as want of heart," is a saying of which the truth is conclusively proved in a sick room. Florence Nightingale says in her "Nursing Notes" that half the suffering that has to be endured is not part of the disease, but the fault of bad nursing. Of course, even in these days of almost endless opportunities we cannot expect everyone to be a good nurse. But as most women are called on at some time or other to undertake nursing it would be as well if all gave some thought to the subject. For instance, a person may not know the right way to change sheets, still if she thought out her own plan before disturbing the patient it would be a great gain to him. As it is two people will come to the bedside and consult each other and the patient, who is first moved to this side, then to that, then up, then down, until by the time the bed is made he is quite exhausted, having, in fact, expended almost enough nerve force to take him a five-mile walk! Often we see untidy sick rooms, even in the homes of the well-to-do, simply because no one thinks of taking anything downstairs. Empty medicine bottles, spoons, plates, and cups are allowed to accumulate until someone with a tray takes all away together. The patient has been worried at first by the confusion, and then is probably awoke out of a refreshing doze by the housemaid clattering about with the tray. Then there is the important subject of food. Why do people seem to lose all ideas of cooking when there is illness in the house? A wife forgets if the husband likes salt, and a mother whether her son takes sugar. Then they have to consult the patient. People choose the hour when one meal is due to talk about the next; all because they do not think.

There are many other points which might be touched upon, such as the banging of doors, talking and laughing just outside the sick room, and forgetting to tell the patient the joke, which he is often quite able to enjoy, keeping sick people waiting unnecessarily for an answer to a message or offering a cup of tea when no kettle boils, are all points to be avoided.

Perhaps the most thoughtless thing of all is the conversation which thoughtless people introduce to a sick room. If a person is ill he is apparently considered to be also deaf, blind, or idiotic. People will turn their faces to the patient and talk in a stage whisper of him. Even when a patient lies apparently unconscious we do not know that he cannot hear. In a cottage a man once lay apparently dying, and as the wife stood ministering to him a neighbour came into the room. She said, in a whisper, "If the Lord takes your old man to-night you won't forget to give mine the job of making his coffin?" The sick man got well, and apologised to the other for not requiring a coffin just then. A doctor relates how he was shown into a room where a man lay dying, and on the staircase which ascended right into the bed-chamber the woman who was leading the way turned to him and remarked, "Mighty awk'ard stairs this to bring a corpse down, 'aint it?" In a word, nothing should be said in a sick room that the patient ought not to hear. All that is necessary must be uttered in a clear low tone, not whispered. "Do unto others as you would they should do unto you" is a rule which, if followed, would ensure the perfection of home nursing.

### Flat Foot Occurring in Nurses.

By Wm. Horrocks, M.B., F.R.C.S., Hon. Surgeon to Bradford Infirmary.

### II.

If the imprint of a natural foot is examined it will be seen that the heel, the pads at the heads of the metatarsal bones, and the tips of the toes touch the ground as the person stands. If the instep is well arched the outer side of the foot does not touch the ground, but usually it rests slightly on this part. In flat foot the whole sole of the foot equally rests upon the ground, and in extreme cases the inner border of the instep is much pressed down, and the outer part is raised. In considering the treatment of this condition the main object is prevention.

All probationers should be especially careful during the early months of their probation, and it is the duty of the superintendent of nurses to advise them as regards their health. Too much care cannot be taken that the probationer has a due allowance of rest and fresh air, that she really goes out when off duty. As far as possible prolonged standing is to be avoided, but when necessary an effort should be made by the probationer to throw her weight on the outer border of the foot, or to stand on the front of the foot and raise the heel slightly from the ground. To assist this without muscular effort it is usual to order boots whose soles and heels are raised a quarter to half an inch higher along the inner than the outer margin. As an additional support the heel is carried forwards on the inner side of the foot to give a firm support to the instep. The boot should be well fastened to the foot, either laced or buttoned, have a wide toe with a straight inner border. The heel should be half or three-quarters of an inch thicker than the sole. If the probationer is not too tired she should be encouraged in "off duty" time to perform exercises to raise the heel, such as dancing or skipping. The feet should be bathed each night in cold or tepid water and well rubbed with a rough towel, not only for purposes of cleanliness but to harden the skin and remove irritating secretions.

If in spite of these precautions flat foot becomes developed to such an extent that the bones become fixed in their abnormal position, it is very questionable whether the probationer is wise in continuing her training, as the work of a nurse is trying to a person in health, and doubly so to one whose every movement is attended with pain.

[Mrs. Magill, lady superintendent to the Bradford Infirmary, kindly read the manuscript, and made the following comments: Flat foot not unfrequently first comes on after a year of training. The treatment recommended above has been fairly successful in alleviating or curing flat foot in those who were so affected. The dietary in the Bradford Infirmary is on the whole fairly satisfactory, but the addition of fresh fruit and more vegetables would be very acceptable. An enclosed space adjoining the hospital where the nurses could play tennis, rounders, &c., would be a great boon to the nurses.]

### Rational Dress in Russia.

The advocacy of rational dress is not apparently left to women reformers in Russia, for a professor of gynecology at Moscow recently read a paper on the subject at the Bordeaux Conference. He found much to commend in the Russian Rational Dress Society, and dwelt exhaustively on the evils attendant on certain forms of clothing.

### Holidays and Health.

[Readers of The Hospital in need of information about health resorts at home or abroad, or desirous of aid in forming holiday plans, are invited to send queries to Editor, 428, Strand, W.C. (marked "Travel" on outside of envelope), which will be answered under this section.]

### THE LAKES.

In old days a visit to the English lakes was an affair of immoderate expense and difficulty. Not to go back to the days of their highest celebrity, days when Coleridge and Wordsworth found the distance of sixteen miles between Grasmere and Keswick insurmountable for ordinary intercourse, it is but a few years since the tour of the Lakes was out of the reach of the "ordinary person," means both of accommodation and locomotion being of the scantiest possible description. Now all is changed. Excursions of the cheapest and most accommodating character can be made from most of the northern manufacturing centres, and "ridin' on Windermere" is a recreation within the reach of every mill-hand. Much has been done, however, to mitigate the nuisance of such an influx of noisy pleasure-seekers to a region hallowed more perhaps than any in England by poetic associations. The railway both at Keswick and Windermere stops well short of intruding on the Lakes, and its place is supplied through the whole district by an admirable service of coaches and chars-à-banc which constitute for most people the very ideal of locomotion. Unpleasing sights and sounds may, it is true, be encountered on the road at the week-end, but the too hilarious tripper seeks his own kind as a rule, and does not wander far beyond recognised centres or care for more exertion than is involved in a short steamer or coach trip. Nothing is easier than to get beyond his range. Everywhere the cheap train service has been followed by a vigorous furbishing up of old inns, the establishment of modern hotels, and the prevalence of a universal condition of lodging-letting among the scanty residents, whereby the once forlorn tourist is enabled to fare very well indeed. The actual season during which there is any pressure on space is a short one, and except at its height prices rule considerably lower than at popular coast resorts.

Leaving the train at Windermere the traveller finds numerous coaches awaiting his choice, notably to Ambleside, Grasmere, or Keswick. For those who desire to live during their stay on coaches and steamers, no better centre can be found than Ambleside, now grown into a bustling and important little season town. At every hour of the day, heavily laden coaches came clattering through the winding street, connecting with every point of interest in the district, and offering combined tours of the most varied description by lake or road at wonderfully moderate rates. Here, too, is ample choice of hotels or lodgings. But to taste the true spirit of mountain solitude and romance, for lonely walks to mountain tarns and pleasant lingerings by still waters undesecrated by bands or "enterprise," Grasmere, Patterdale, or Rossthwaite will be a happier choice. Grasmere must always stand first in interest for the lover of Wordsworth. Something of the restful sense experienced at that grave by one of our truest living poets hangs still about the little village:—

> "Here one may scarce believe the whole wide world

> Is not at peace, and all man's heart at rest."

A turn of the road and all the habitations are lost to sight, the mountains close round in lofty solitude, and every sound dies away except that ceaseless murmur which follows the wayfarer whither he goes, the same which haunted the mariner in his waking dream.

> "A noise like of a hidden brook
> In the leafy month of June,
> That to the sleeping woods all night
> Singeth a quiet tune."

To return to more prosaic matters, Grasmere has good store of accommodation for man and beast, but though her cottages are wonderfully elastic her limit is soon reached, and it is well either to make sure of a room beforehand or to arrive early enough in the day to make it possible to move on elsewhere in case of failure. Mrs. Robinson, whose husband is the well-known scarlet-coated John of the Windermere and Grasmere coach, has a well-appointed house just on the outskirts of the village, and is prepared to make special terms for nurses needing rest, except just in the month of August, when terms rule high. From Grasmere a wonderful coach drive over the mountain pass of Grisedale Hause (1,929 ft.) leads to the Valley of Patterdale, through which the little Goldrill runs its pleasant course to its home in Ulleswater. Wood's private temperance hotel at the head of the lake offers excellent accommodation and very reasonable terms "en pension." Here also, except in August, nurses will be welcomed on reduced terms. It is an ideal spot, this upper end of Ulleswater, from which anyone might be loth to stir in restless search of further beauties. Steamers to Pooley Bridge, whence a coach runs to Penrith, form the connecting link on the northern side with the railway. The energetic may make an easy ascent of Helvellyn from this point, and endless less arduous excursions by boat or on foot lie within reach.

For the pedestrian who abhors steam and scorns even the coach, a good centre is the less well-known village of Rossthwaite, situate on a noisy little tributary of the Derwent in Borrowdale. Within the compass of a dozen miles are innumerable notable points, embracing the ascent of Skiddaw, of Scaw Fell, of Langdale Pikes, and Helvellyn. Prices here, too are very moderate.

One word about weather in Lake-land. It would certainly not be judicious to go that way without an umbrella, but it is a great mistake to go cumbered with heavy waterproofs, treating the rain as a deadly enemy. It has its days for descending gently, generously, patiently—the proper attitude is then not despair, but defiance. Put on a skirt which will not spoil and a light jacket or short cloak, and go out prepared to enjoy what comes; then if you return tingling with exertion, even though with soaked feet and moist garments, you will find this little essay at "kneipping" by no means an unhealthful experiment. But the thing to be avoided at all costs is sitting on a damp coach wrapped up, and safe as you think in an unventilated mackintosh or waterproof until the blood is congealed, the cheeks pale, and life begins to feel no longer worth living. This is the sort of thing which leads to neuralgia, a very real trouble to residents in this region. As for the gracious rain which breaks out at any moment of almost every day, only to pass gaily away over the mountains, this may be safely ignored; in ten minutes the roads are as though no rain had fallen, and no dress should be worn likely to resent a sprinkle of this nature.

### Everybody's Opinion.

[Correspondence on all subjects is invited, but we cannot in any way be responsible for the opinions expressed by our correspondents. No communications can be entertained if the name and address of the correspondent is not given, or unless one side of the paper only be written on.]

### PRIVATE NURSES AND PRESENTS.

"A Private Nurse of Ten Years' Experience" writes: I cannot let the paragraph, "Can This be True?" in "The Hospital Nursing Supplement" for the 17th inst., pass without replying at once. Surely not, and I hope other first-class nurses will quickly contradict the statement as to presents. During a varied experience, chiefly amongst upper middle class and the nobility, on one or two occasions only has an honorarium ever been offered and gently but firmly declined. I number among my firmest friends patients from whom I have never accepted anything beyond just fees, and am sure many other self-respecting nurses will testify the same. As long as so-called "Nursing Homes and Institutes" take women without hospital training, and after letting them see and assist with patients for a few months, send them out as trained nurses, paying them very small salaries, but receiving for them full, high fees, instances will occur and disgrace our profession.

[We agree with the sentiments of our correspondent, and admire her self-respecting attitude. We fear that untrained nurses are not the only ones who act differently, and we learn with regret that employers of those whom our correspondent calls "first-class nurses" find that presents are more often accepted than declined.—Ed. *T. H.*]

### A Nurse's Winter in Canada.

### FIRST IMPRESSIONS.

In preparation for a visit to a snow country I naturally thought that I should need warm underclothing, and as I was going to winter in Montreal, set up an abundant supply of Scotch wool. It is still at the bottom of my "Saratoga," and must remain there until it is required in some milder climate than this. It sounds paradoxical, but in Canada as in America, houses are kept at a high temperature, hot pipes encircling the rooms and the passages at the same even heat. An Englishwoman feels at first as if she could hardly breathe in such heat night and day, double windows excluding any chance of a draught. I shall, I believe, feel the cold much more a winter or two hence, and probably be just as indignant as any Canadian when Jean Baptiste, the stoker, lets the temperature of the house drop 10 degrees during his slumbers. But at present cobweb merino has replaced Scotch wool, and my stockings are of the thinnest thread, and I wear one light underskirt. Tweed dresses are a mistake, so mine are put in camphor, and I have purchased a cashmere of light texture.

The preparation to go out of doors is rather a lengthy performance, and perhaps this is why Canadians adopt telephones in private houses. There is no necessity to go marketing in Montreal—you turn the "crank," and converse with the butcher and greengrocer whilst completing your toilet.

For outdoor use I have a pair of "over-stockings," as thick as Scotch shooting ones, drawn over thin house slippers; then come the "rubbers," which fit tightly over the house slippers and stockings; next in order come capacious "bloomers," resembling the cycling garments of the modern woman. Mine are made of the thickest flannel, and if any skirt is worn it is tucked inside them. Not possessing furs, I don my "blanket coat," a white blanket made up into a wide ulster, and lined with flannel, red borders round the skirt and hood, make a picturesque bit of colour in the snow, and a knitted scarf is tied round the waist. For tobogganing a knitted stocking cap, like a Neapolitan fishermen's cap, goes over hair and ears, as the hood of the blanket ulster, which is drawn up like a monk's hood, is apt to blow off, and neuralgia would follow.

Luggage is much more conveniently dealt with in this country than at home. At the ticket station before the terminus, agents for the company employed look at the number of your checks, of which one is given for each piece of luggage, which has a corresponding number on it. The safety of these checks being all the responsibility the traveller has regarding the welfare of the wardrobe, they are not parted with until all baggage is safely deposited in one's own room. For a single woman travelling across the Continent much worry is escaped by this system. A quarter is the charge for each piece of baggage, and it was not until I had paid the round sum of 5s. for my five pieces of luggage that I understood the *raison d'être* of the large American trunk which labels the nationality of its proprietor, for there is no restriction as to size.

My first glimpse of Montreal in her winter costume was fascinating. When I got outside the railway "depôt" I expected to see plenty of closed conveyances ready to shelter me from the intense cold, but (curious custom for a cold climate) only open sleighs were used. There were rows and rows of these for hire with a delightful jingling of bells. All public sleighs are compelled to carry them for the safety of the public during the otherwise noiseless passage through the snow. Public sleighs have a delightful abundance of fur "robes" or carriage rugs, of oppossum or bear. One is for sitting on and hangs over the back of the sleigh, and two or more to be wrapped round the body. I was rather anxious as to the charge which might be made for this luxurious drive; but as I did not know my way, and the driver spoke only French (which is not the French of old France, but of new), I did not attempt to question him, but gave myself up to the enjoyment of my first sleigh drive. He tucked me well up, jumped quickly to his high box seat, and away we flew over the dazzling white snow, with a deep blue sky above. On reaching my destination I was pleasantly surprised by the driver charging only a "quarter."

Breakfast was just over next morning when Canadian hospitality declared itself. I had posted a letter of introduction, and by ten a.m. I was carried off bag and baggage in a magnificent sleigh of polished bird's-eye maple, and almost smothered in beautiful buffalo robes. I could hardly believe that two weeks before I had been hard at work in my ward in London, looking on to a prospect of fog, rain, and slush! One of the commonest operations in Western hospitals during the winter season is the amputation of frozen toes and feet. A friend of mine had to part with half his right foot, his toes having been neglected till mortification set in.

During the ice carnival one of the house party insisted on wearing what her brother called her "New York" hat in place of her less dressy fur one. I tried to dissuade her as there was a wind blowing—a wind with a temperature 30 below zero is a thing to beware of. The girl had short hair and nothing protected her ears. In the excitement of the procession, whilst a car of McGill College students was passing, an acquaintance called out, "Nellie, your ear is frozen!" (I have seen since then a total stranger stop a timid old lady with the remark, "Pardon me, madam, but your nose is frozen.") By gently rubbing Nellie's chalk-coloured appendage with soft snow the spark of life in it was revived. The thawing stage was of course very painful, but the girl's one thought was, "Oh, dear, what shall I do at the carnival ball to-night? My ear will be at the raw beef stage." But she enjoyed her ball all the same, and I wish I could tell you of all the entertainments which fell to my lot that first winter. Suffice it to say that although I have to wait for my coffee until milk is melted in the morning, and dare not open my windows to let the new year in or the old year out as at home, still Canada has its advantages. I can speak to my sister, the busy matron of a hospital in Toronto, through the long distance telephone and wish her a merry Christmas. And I wish in old England, worn and grey, there were such merry hearts as those of hospitable Canadians.

### Notes and Queries.

### Queries.

(229) *Noma or Unna.*—Is the paste described last week under the first name the same as Unna's mixture?—*Inquirer.*

(230) *Age.*—Can you tell me of any metropolitan or provincial hospitals where probationers are taken under 20 years of age?—*N. C.*

(231) *Abroad.*—Please inform me whether "Burdett's Directory" will give me information about hospitals in India or America. I want to go out as assistant nurse.—*Union.*

(232) *Unsweetened Milk.*—Where can I obtain this in tins?—*Kath.*

(233) *Putty Powder.*—Can you tell me what this is composed of?—*N. H.*

### Answers.

(229) *Noma or Unna* (*Inquirer*).—It should have been given as "Unna." This is one of many instances when indistinct writing has caused an annoying blunder.

(230) *Age* (*N. C.*).—We constantly answer this question—as you will see by looking back through our columns. We do not know of any training schools where such young probationers are taken, and we are of opinion that it would be most undesirable for them to do so. If a children's hospital in the provinces would suit, you could get addresses from "Burdett's Hospital Annual."

(231) *Abroad* (*Union*).—The directory is not yet published. "Burdett's Hospital Annual" of 1894 will help you. America now trains plenty of nurses, therefore an assistant nurse from England could not get a hospital post there. See "Answers to Queries," Nos. 135 and 138, in The Hospital of May 11th, 1895.

(232) *Unsweetened Milk* (*Kath*).—See page 314, The Hospital, August 3rd, 1895.

(233) *Putty Powder* (*N. H.*).—Putty powder is the binoxide of tin. It is prepared from the scum or oxide which forms on the surface of melted tin. This is purified by calcination, and then ground to powder. Putty powder is used for polishing stone, glass, and other substances; also in giving glass an opaque white colour.

### The Book World for Women and Nurses.

A Healthy Home. By Francis Vacher. (Published at the office of the *Sanitary Record*. Price 3s. 6d.)

The air is full of sanitation. Everyone talks about it and considers himself or herself—for women are well to the fore in this case—an authority on the subject. The result of this craze is that hardly a week passes without the appearance of some text-book on sanitation, and we confess to feeling some slight curiosity as to the kind of *réchauffé* served up in each new volume. Dr. Francis Vacher states that his object in publishing "A Healthy Home" is to furnish as much information as is needed for the guidance of anyone wishing to build a dwelling-house who has neither time, nor inclination, nor the preliminary technical knowledge to study effectually the larger text-books that exist. This little book professes to condense into a nutshell, as it were, all the information which every man or woman should have before embarking on an unknown sea of bricks and mortar. We must say at once this object is carried out extremely well. In a volume of 204 pages is to be found all that is necessary and little that is superfluous. Beginning with site, soil, and aspect of the house, the author next gives an account of various building materials, such as bricks, slates, tiles, &c., briefly describing how each is made, which is best for special purposes, and how to detect defective materials. Then follow chapters on construction, management, warming, lighting, ventilation, and decoration and furniture; nor are stables and cowsheds omitted. A most useful chapter is that on the "Obligations of Householders," which explains such mysteries as rates and taxes, conditions of tenancy and leases, and also quotes some useful sections from the Public Health Act. We could have wished that these subjects had been dealt with in greater detail, and the chapter on "How to Keep the House Clean" omitted. Directions about turning down and airing beds, washing bedroom crockery, shaking door-mats, polishing handles and knockers and cleaning grates, and such general housemaid's work are quite superfluous here. A householder who has still these things to learn requires more detailed instruction than can be crammed into a dozen pages, and he who does know already will find nothing new or original in it. With this exception the book is excellent, and will we hope, be widely read. If everyone possessed all the useful knowledge it contains the "jerry builder" would soon be extinct.

Text-Book of Anatomy and Physiology for Nurses. Compiled by Diana Clifford Kimber. (Macmillan and Co., New York and London. 1894.)

The predominant feeling on first opening this book is a gentle shock of surprise at the profundity of knowledge required in a properly trained nurse. No doubt this is partly due to the illustrations which, scattered in large profusion throughout its pages, suggest a minuteness of anatomical and histological teaching which one hardly expects average nurses to receive.

Reference to the text, however, shows that full as the book may be it really does not go further than, if indeed so far as, is common enough in lectures and in other works of much more humble appearance.

It may, however, be doubted whether it is worth while for nurses to enter into such details as are given in some of the illustrations. The pictures of the palate bone, the inferior turbinate bone, the superior maxilla with all the muscular attachments, &c., must be infinitely puzzling to the careful student who searches for the meaning of all things and finds no words of explanation, and the section of the female pelvis, covered as it is with very illegible names which are nowhere mentioned in the text, must raise much unanswered questioning. The work itself is thoroughly readable and, so far as a not too critical examination tells us, accurate. Every part seems to have been culled from one or other of several most trustworthy sources, and the student who reads it carefully cannot fail to obtain a considerable insight into the uses and construction of the various organs of the body. At the same time we are struck by a certain want of proportion in the arrangement of the mental food, good as each morsel may be, offered for the consumption of the budding nurse. It is definitely more anatomical than physiological, and herein lies its weakness. Physiology can afford to be vague, anatomy cannot. To say that a nerve transmits an impulse, or that a muscle responds to such an impulse, is doubtless a crude statement and does not attempt to go to the bottom of things, but at least it conveys the idea which was intended. With anatomy it is different; no idea except one of confusion is conveyed by the statement that "the muscles of mastication are the masseter, the temporal, and the external and internal pterygoid. They all have their origin in the immovable bones of the skull, and are all inserted into the moveable lower jaw." This is all absolutely correct, but it gives no notion of the action of these different muscles, and but little object is gained by mentioning them separately unless the student is made to understand the part played by each. The difficulty of teaching anatomy is far greater than many people would imagine, especially when there is not room to be precise. To show how the book drifts to the anatomical rather than the physiological side, we may draw attention to a curious lapse in the section on the nervous system where, although there are admirable illustrations of nerve structures, cells, fibres, spinal cord, nerve endings, tactile corpuscles, &c., there is no mention in the index of reflex action, and, as far as we could find, no reference to it in the text except that in describing the condition found after division of the spinal cord it is said that "this power of transforming an afferent into an efferent impulse is termed the reflex power of the spinal cord."

The book is admirably got up; the paper, the printing, and many of the illustrations are exceedingly good. We cannot, however, but feel that while in parts there is even a surplusage of information there are here and there *lacunæ* which would leave an untaught reader full of doubt.

### Appointments.

[It is requested that successful candidates will send a copy of their applications and testimonials, with date of election, to The Editor, The Lodge, Porchester Square, W.]

West Ham Union Infirmary.—Miss Constance Prichard has been appointed Matron of this infirmary. She was trained at St. Bartholomew's Hospital, 1884 to 1887; on staff till 1889. Miss Prichard was sister of ward at Newcastle (Fleming Memorial Hospital) in 1889-1890; night superintendent, West London Hospital, 1890-1892; and superintendent of nurses at Tottenham Hospital, 1892-1895.

Lloyd's Infectious Hospital. Altrincham.—Miss E. Winstanley has been made Nurse-Matron of this hospital. She was trained at the Manchester Royal Infirmary and remained there and at the Convalescent Hospital at Cheadle for six years and a-half. Miss Winstanley afterwards worked as district nurse at Dukinfield for a year and eight months and then did private nursing. She holds very good testimonials, and many good wishes accompany her to her new work.

Royal Sea Bathing Infirmary, Margate.—Miss E. Carew Oxley has been appointed Matron of this hospital. She was trained at Guy's Hospital, and was afterwards sister under Mr. Arthur Durham in male surgical ward and sister in female medical and children's ward. Miss Carew Oxley was lady superintendent in the Indian Nursing Service, Madras Presidency, and matron of St. Bartholomew's Hospital at Chatham, and her excellent testimonials show her to be possessed of considerable administrative ability as well as being an accomplished nurse. We wish Miss Carew Oxley a successful career in the new sphere which her varied experience has fitted her to fill with distinction.

## Figures and Tables

**FIG 1 f1:**
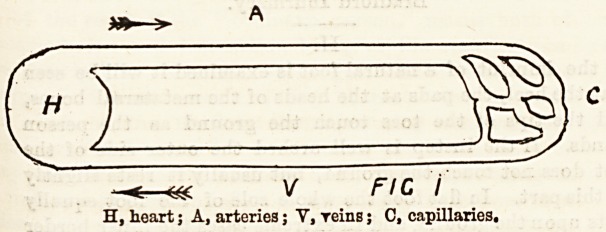


**FIG 2 f2:**
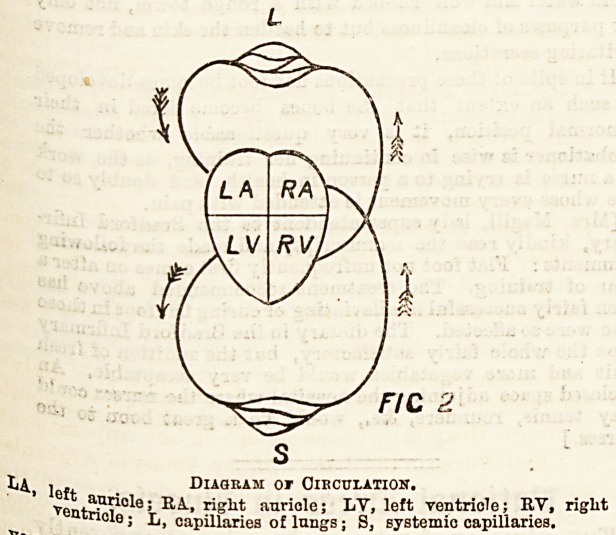


**FIG 3 f3:**